# Genome-wide survey of post-meiotic segregation during yeast recombination

**DOI:** 10.1186/gb-2011-12-4-r36

**Published:** 2011-04-11

**Authors:** Eugenio Mancera, Richard Bourgon, Wolfgang Huber, Lars M Steinmetz

**Affiliations:** 1 European Molecular Biology Laboratory, Meyerhofstrasse 1, 69117 Heidelberg, Germany; 2 European Molecular Biology Laboratory, European Bioinformatics Institute, Cambridge CB10 1SD, UK; 3 Genentech, Inc., 1 DNA Way, South San Francisco, CA 94080-4990, USA

## Abstract

**Background:**

When mismatches in heteroduplex DNA formed during meiotic recombination are left unrepaired, post-meiotic segregation of the two mismatched alleles occurs during the ensuing round of mitosis. This gives rise to somatic mosaicism in multicellular organisms and leads to unexpected allelic combinations among progeny. Despite its implications for inheritance, post-meiotic segregation has been studied at only a few loci.

**Results:**

By genotyping tens of thousands of genetic markers in yeast segregants and their clonal progeny, we analyzed post-meiotic segregation at a genome-wide scale. We show that post-meiotic segregation occurs in close to 10% of recombination events. Although the overall number of markers affected in a single meiosis is small, the rate of post-meiotic segregation is more than five orders of magnitude larger than the base substitution mutation rate. Post-meiotic segregation took place with equal relative frequency in crossovers and non-crossovers, and usually at the edges of gene conversion tracts. Furthermore, post-meiotic segregation tended to occur in markers that are isolated from other heterozygosities and preferentially at polymorphism types that are relatively uncommon in the yeast species.

**Conclusions:**

Overall, our survey reveals the genome-wide characteristics of post-meiotic segregation. The results show that post-meiotic segregation is widespread in meiotic recombination and could be a significant determinant of allelic inheritance and allele frequencies at the population level.

## Background

**Figure 1:**
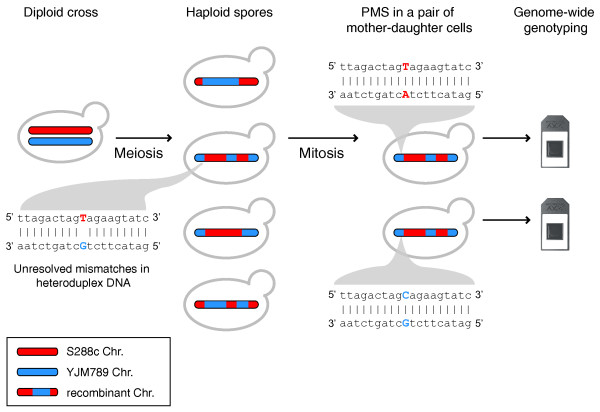
**Genome-wide post-meiotic segregation mapping**. Schematic description of the approach to map post-meiotic segregation (PMS) genome-wide. The four pairs of mother-daughter cells resulting from the first mitosis of each spore were genotyped using a tiling microarray.

In sexually reproducing organisms, homologous chromosomes exchange genetic information through meiotic recombination. This process, which occurs in most eukaryotes, is an important determinant of allelic variation [1,2]. Recombination is triggered by the formation of programmed double-strand breaks (DSBs), which are typically repaired using the homologous chromosome as a template. Meiotic DSB repair often produces regions of gene conversion, which may or may not be accompanied by a reciprocal exchange of homologous chromosomal arms, thereby producing crossovers (COs) and non-crossovers (NCOs), respectively [3]. The pairing of a single strand from one homolog with the complementary strand from the other produces heteroduplex DNA with mismatches at heterozygous positions. Repair of these mismatches results in either gene conversion or restoration of the original genotype. If the mismatches are not repaired, both alleles will persist in the meiotic product and will segregate during the first mitotic division (Figure 1). This phenomenon, known as post-meiotic segregation (PMS) [4], has the potential to cause somatic mosaicism in multicellular organisms, since the two cells resulting from the first zygotic division will possess different alleles [5]. Moreover, if the somatic lines are genetically different from the germ line, PMS will lead to unexpected allelic combinations among progeny. As a consequence, simple traits determined by such a locus may appear to follow complex inheritance [5].

Despite its implications for inheritance, PMS has been previously investigated mainly on a locus-by-locus basis ([4,6-9] and references in [4]). The difficulty of studying PMS comes from the fact that its detection requires scoring genetic markers in the eight cells resulting from the first mitotic division of each of the four meiotic products. Filamentous fungi generating eight ascospores as a result of an extra post-meiotic mitotic division during sporulation have therefore often been used to study PMS at isolated loci [10-12]. Fission and budding yeast have also been models for PMS because the occurrence of PMS in markers conferring a phenotype causes colonies grown from a single spore to be sectored [13,14]. Previous genome-wide studies of meiotic recombination, all performed in budding yeast, have surveyed colonies of cells arising from each meiotic product [15-17]. In such colonies PMS results in allelic mixtures that are challenging to genotype. One study employing next generation sequencing only confirmed one PMS case out of five putative events in a single analyzed tetrad [17]. Thus, little information exists about the genome-wide frequency and characteristics of PMS in any organism. Here, we achieved genome-wide characterization of PMS in *Saccharomyces cerevisiae *by simultaneously assessing over 52,000 heterozygosities in mother and daughter cell pairs of all the products of several meioses (Figure 1). PMS events were observed in close to 10% of recombination events, occurring with equal relative frequency in COs and NCOs, and mostly at the ends of gene conversion tracts. Moreover, markers where PMS occurred tend to be more isolated than other markers and are mainly SNPs of specific types. Our approach allowed genome-wide detection of this elusive genetic phenomenon and shows that PMS could be an important determinant of allele frequencies at the population level.

## Results and discussion

To survey PMS genome-wide we first dissected tetrads obtained from a cross between two diverged yeast strains - a laboratory strain, S288c, and a clinical isolate, YJM789 [18,19]. These strains were selected due to their substantial genetic diversity. In wild populations, including those of *S. cerevisiae *[20,21], most individuals are heterozygous and the S288c/YJM789 cross may therefore resemble conditions in the wild closer than homozygous strains. Although the large number of polymorphisms between the strains allows high-resolution genotyping, heterozygosities could also affect meiotic recombination [22]. Nevertheless, in the S288c/YJM789 cross, the genomic distribution of recombination events has been shown not to be markedly perturbed [15,16]. It has also been observed that certain allelic combinations of the mismatch repair (MMR) genes are incompatible, leading to elevated mitotic mutation rates in segregants of intra-species yeast hybrids. Strains with an S288c allele of *MLH1 *in combination with the SK1 (another *S. cerevisiae *strain) allele of *PMS1 *show an approximately 100-fold higher mutation rate in the *lys2-A14 *mutator assay [23]. This observation is consistent with the central role that *MLH1 *and *PMS1 *play in MMR. YJM789 carries the ancestral form of both genes and is therefore compatible with S288c and SK1. Thus, we do not expect the progeny of the S288c/YJM789 cross to show elevated mutation rates [23,24].

We allowed each of the dissected spores to germinate and divide mitotically, and then separated the two resulting cells under a dissection microscope (Materials and methods). The four pairs of mother and daughter cells arising from each tetrad were genotyped using tiling microarrays and a supervised modality of the *ssGenotyping *algorithm [25], trained on a large set of published data [16]. A total of four tetrads were analyzed. Markers where PMS occurred (PMS markers) were identified by comparing the genotypes from mother and daughter cells in each pair (Figure 1). For each identified PMS event in the two tetrads with the most events, conventional Sanger sequencing was performed as validation, and no false positives were discovered.

**Figure 2:**
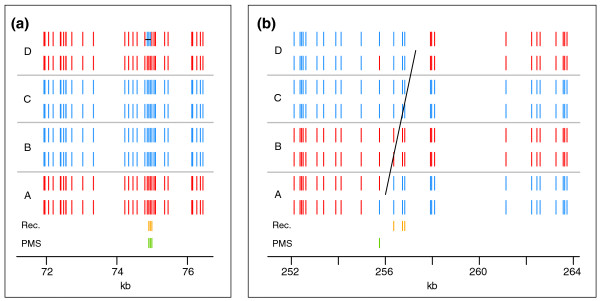
**Examples of post-meiotic segregation**. **(a,b) **Close-ups of a NCO in chromosome VI (a) and a CO in chromosome XVI (b) containing markers where PMS occurred. Red/blue vertical segments represent markers with the S288c/YJM789 genotype along the chromosomes of the two mother and daughter cells resulting from the first mitosis of each spore (A, B, C and D). The horizontal black line indicates the inferred NCO, and the diagonal, the CO. Green vertical segments immediately on top of the coordinate axis denote markers where PMS occurred and orange segments denote markers with non-Mendelian segregation.

Among the four tetrads, we found a total of 52 markers where PMS occurred (18, 6, 17, and 11 per tetrad; Additional file 1). This constitutes 1.2% of the overall number of markers involved in recombination events (Additional files 2 and 3). There were four instances in which PMS occurred in more than one marker in the same recombination event (for example, Figure 2a). PMS events were present in more than 9% of the overall recombination events: 46 of the total 499 COs and NCOs had at least one marker exhibiting PMS (Additional file 3). Furthermore, COs containing no converted markers presumably correspond to recombination events in which heteroduplex DNA contained no polymorphic positions, and which therefore could not produce gene conversion or PMS. In fact, the inter-marker spacing at the flanks of these COs was considerably larger than a typical inter-marker interval (median inter-marker spacing of 2.1 kb versus 78 bp). If such COs are set aside, the portion of recombination events with at least one PMS marker increased to 10.6%. The high number of recombination events where PMS occurred across the genome indicates that PMS is a widespread phenomenon in recombination and a significant contributor to allelic diversity during meiosis.

Although the MMR machinery that resolves mismatches during the formation of COs or NCOs is thought to be the same [4], it has been observed that a fraction of COs presents higher PMS frequencies [26]. Whether PMS occurs more frequently in COs overall or in NCOs has not been tested. Out of the 46 PMS events, 28 occurred in COs and 18 in NCOs. Notably, this ratio did not significantly differ from the overall genomic CO to NCO ratio observed (336 COs:163 NCOs; Additional file 3; Fisher exact test, *P *= 0.33). Thus, our data do not suggest that the efficiency of the MMR machinery depends on whether the heteroduplex is resolved towards a CO or a NCO.

Interestingly, we observed that markers where PMS occurred tended to be at the ends of gene conversion tracts (Figure S1 in Additional file 4). Only six PMS events were not at the end of a tract. To test whether this observation statistically deviates from a scenario in which PMS occurs uniformly along conversion tracts, we focused on the 26 tracts containing at least one PMS marker and consisting of three or more markers. (Tracts smaller than three markers have only terminal markers.) Among these 26 events, there were 20 (76.9%) with a terminal PMS marker, and together they contained 32 PMS markers, of which 22 were terminal. If we assign 32 PMS events uniformly at random to this set of events, the probability of seeing such a high fraction of events with a terminal PMS marker is <0.001 (Figure S2 in Additional file 4; Materials and methods). This provides strong evidence that PMS occurred predominantly at terminal markers.

**Figure 3:**
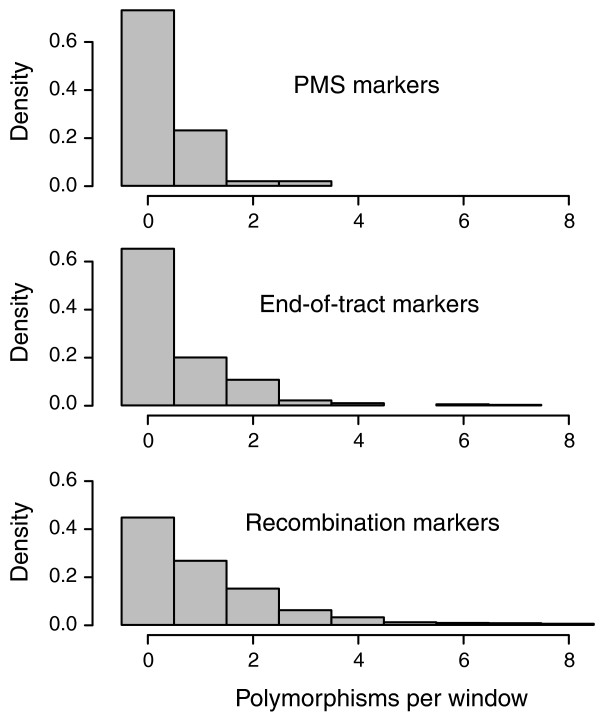
**Post-meiotic segregation markers are relatively isolated from other polymorphisms**. Histograms showing the marker density in 100-bp windows centered on PMS markers (upper panel), centered on markers located at the end of conversion tracts (middle panel), and centered on overall markers in recombination intervals (lower panel). A range of window sizes produced qualitatively similar results. The median distance to the nearest polymorphism for markers at the end of conversion tracts was 58 bp larger than for all markers in recombination events (Wilcoxon test, *P *< 0.0001) and the median distance to the nearest polymorphism for PMS markers was 49 bp larger than for all end-of-interval markers (Wilcoxon test, *P *= 0.002).

It has been previously shown that neighboring polymorphisms influence the PMS frequency of a given marker [27,28]. To investigate the effect of surrounding heterozygosities, we first considered the polymorphisms around PMS markers independently of whether they also showed PMS. We found that 100-bp windows centered on the PMS markers were twice as likely to not contain any other polymorphism as windows centered on markers not showing PMS (Figure 3, compare top and bottom panels; Fisher exact test, *P *= 2.5 × 10^-10^). A range of other window sizes (50 to 300 bp) gave qualitatively similar results. Since the ends of gene conversion tracts tend to have lower marker density (Figure 3, compare middle and bottom panels), the preferential position of PMS markers at the end of tracts might have been the cause of the observed relative isolation of PMS markers. This turned out not to be the case: the median distance to the nearest polymorphism for PMS markers was 49 bp larger than for all end-of-interval markers (Figure 3; Wilcoxon test, *P *= 0.002). Thus, PMS markers appear to be better separated from neighboring polymorphisms than would be expected by chance, even given their positioning at the end of conversion tracts. This suggests that the MMR machinery may be more responsive to heteroduplex regions with a higher density of mismatches.

The MMR machinery repairs mismatches by excising a segment of one of the two single strands, often as large as 900 bp [27]. Therefore, adjacent mismatches, if present within the excised fragment, can be co-repaired. If MMR repair takes place over large tracts of heteroduplex DNA - that is, if repair does not take place one mismatch at a time - then it is also conceivable that tracts of heteroduplex DNA that contain multiple mismatches may be left unrepaired. In our data, consecutive PMS markers in the same conversion tract may provide evidence of this. Altogether, one recombination event involved two PMS markers, and two involved three (Figure 2a; Figure S3 in Additional file 4). Remarkably, markers where PMS occurred in the same conversion tract were always adjacent to each other, with no other polymorphisms in between (Figure 2a; Figure S3 in Additional file 4). Among these, the shortest distance between neighboring PMS markers was 43 bp, and the longest was 488 bp. All of these events were at the end of a conversion tract. Having established that a high fraction of the observed PMS events occurred in the final marker of a recombination tract, we next asked if the observed end-of-tract multi-marker PMS events were likely to be mechanistically linked or were rather due to chance co-localizations of independent PMS events. Using, as before, the 26 tracts with three or more markers that were observed to contain a PMS marker, we ran a second simulation. This simulation included end-of-event bias: simulated PMS markers were assigned to internal and terminal positions in proportions similar to those observed in the actual data (see Materials and methods). In this second simulation, the probability of seeing three or more recombination events with end-of-tract multi-marker PMS events is very unlikely (*P *< 0.001). This suggests that the occurrence of PMS in a given marker increases the frequency of PMS in the surrounding markers, at least for terminal PMS events. This finding is consistent with previous observations made at the budding yeast *HIS4 *locus [27].

In our whole dataset, we observed only one instance in which two different spores had PMS in the same marker. Both of these PMS events were located in the two spores involved in a single CO, resulting in 4:4 aberrant segregation (Figure 2b). Such a pattern of symmetric heteroduplex tracts is expected to be the result of branch migration of a Holliday junction during DSB repair. Aberrant 4:4 segregation resulting from symmetric heteroduplex DNA was one of the original predictions of the Holliday model of recombination. However, since aberrant 4:4 segregation is rarely observed in *S. cerevisiae*, Holliday junctions are currently thought to be resolved before branch migration [6]. The rare cases of observed aberrant 4:4 segregation have been alternatively explained as the result of two independent recombination events involving all four chromatids [6]. Although the event observed here has a complex topology (Figure 2b), the fact that only two chromatids show recombinant markers suggests that it resulted from symmetric heteroduplex tracts during the repair of a single DSB.

Having explored the context in which PMS markers are located in terms of other polymorphisms, we next considered the types of polymorphisms where PMS occurred. Insertions or deletions (indels) accounted for 9.4% of the polymorphisms in gene conversion regions, a similar proportion to that of indels present between the whole genomes of S288c and YJM789 (approximately 9.0%) [29]. Of the markers where PMS occurred, 98.1%, or all but one (a 29-bp indel), were SNPs. If one treats the 52 PMS markers as independent Bernoulli draws from the pool of markers involved in a recombination event, then the chance of drawing 0 or 1 indels is 0.03. However, given the preferential occurrence of PMS at the ends of conversion tracts, if only such positions are considered, the fraction of indels drops to 6.4%, and the probability of observing 0 or 1 indels in 52 events rises to 0.15. Previous work has shown that the MMR machinery has similar binding affinities to 1-bp indel mismatches as to the strongest bound SNP mismatch [30]. Other indel mismatches have been observed to be bound with lower affinity than 1-bp indels [30]. Furthermore, null mutations in the main MMR proteins have been observed to exert a similar effect in the repair frequency of SNP and small indel mismatches [4]. From our genome-wide PMS data we cannot conclude - with statistical significance - whether indel mismatches are better repaired than SNP mismatches.

**Figure 4:**
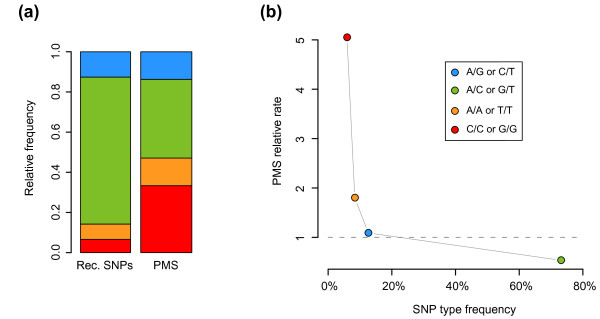
**Post-meiotic segregation occurs preferentially at specific polymorphism types**. **(a) **Relative frequencies of the possible mismatches given the SNPs found in PMS events and in recombination events (Rec. SNPs). **(b) **Inverse relationship between the frequency of the different SNP types in the *S. cerevisiae *species and the efficiency with which the mismatches generated by the SNPs are repaired (PMS relative rate = PMS frequency/Recombination SNP frequency). In the figure the frequencies of SNPs between S288c and YJM789 are shown. The result is qualitatively the same when calculating SNP frequencies with other strains [37].

To gain further insight into the sequence characteristics of PMS events and their evolutionary hallmarks, we focused on SNPs and analyzed the type of bases that are involved in PMS. Any given SNP can give rise to two possible mismatches, depending on which base is resected during recombination. As shown in Figure 4a, at markers where PMS occurred, we observed SNPs that could generate all possible mismatches (Additional file 1). However, the relative frequencies of SNP types at PMS markers differed strongly from those of all SNPs found in recombination events (Figure 4a; Fisher exact test, *P *= 4 × 10^-9^). SNPs that generate C/C or G/G and A/A or T/T mismatches are, respectively, 5.0 and 1.8 times more frequent in PMS events than in overall recombination events. On the other hand, SNPs giving rise to A/G or C/T mismatches are approximately as frequent as in recombination events, and SNPs producing A/C or G/T mismatches are only half as frequent. These deviations in the relative frequencies do not seem to be caused by the preferential occurrence of PMS at the end of conversion tracts, since the different SNP classes are uniformly distributed along tracts (Figure S4 in Additional file 4). We thus find clear differences in the genome-wide PMS rates between all four SNP classes.

The enrichment of SNPs generating C/C or G/G mismatches is a likely reflection of the known relative inefficiency of C/C repair [31,32]. At the *ARG4 *and *HIS4 *loci, C/C repair has been reported to be between three- and five-fold less efficient than the repair of other mismatches [7,8]. Similar efficiency reductions have been found in other fungi (*Schizosaccharomyces pombe*) [33], in animals [34] and in prokaryotes [35]. It has even been proposed that C/C mismatches are repaired by a different molecular machinery than other mismatches [36]. It is also known that the best-repaired mismatch is G/T. Binding studies *in vitro *have revealed that the MSH2-MSH6 complex, a central player of MMR, has the highest affinity to G/T mismatches [30,34]. The efficiency with which other mismatches are repaired is less clear, especially *in vivo*. A/A and T/T mismatches, for example, have been reported to be repaired less efficiently in mitotic assays [31], but also as efficiently as other mismatches during meiosis [7,9]. Here we find clear differences in the genome-wide PMS rate between all four SNP classes (Figure 4a), suggesting that each mismatch class is repaired with a different efficiency *in vivo*.

Interestingly, the repair efficiency of mismatches observed here was inversely related to the overall frequencies of the associated SNP classes in *S. cerevisiae *(Figure 4b). This was not only true for SNPs between S288c and YJM789, but also for SNPs among several recently sequenced yeast strains [37]. The distribution of SNP classes in the population reflects, at least in part, the frequency with which the MMR machinery encounters the mismatches caused by such SNPs. The fact that the mismatches associated with the most common SNP classes are also the most efficiently repaired may therefore be a consequence of selective pressure favoring removal of mutation-associated mismatches. MMR protein variants that are better at repairing common mismatches would be selected for. There is support for this hypothesis in *Escherichia coli*, where the frequency of different DNA polymerase III errors *in vitro *is positively related to the repair efficiency of mismatches in phage genomes [38]. The same category of SNPs that is most numerous in the budding yeast genomes is also the only one to form purine/pyrimidine mismatches. Therefore, it may indeed be the case that the MMR machinery has evolved to more readily recognize such mismatches.

## Conclusions

To our knowledge, this work is the first genome-wide survey of PMS in any organism: traditional studies on PMS have focused on a small number of genetic markers in many meiosis; we, on the other hand, have examined tens of thousands of heterozygosities along the genome in a smaller number of meioses. Our work takes previous genome-wide recombination studies [15-17] one step further by analyzing not only the products of meiosis, but also the products of the first mitotic division of each spore. We show that, in terms of genome-wide recombination events, PMS is widespread and preferentially affects SNP types that are relatively rare within the species, as well as SNPs that are isolated and at the ends of conversion tracts. Although PMS occurred in only a small fraction of markers, the number of bases affected per meiosis is considerably larger than those altered by base substitution mutations. Taking into account only the 48,933 genotyped markers consisting of single SNPs in the S288c/YJM789 cross, we observed 51 SNPs affected by PMS in 16 spores. Thus, the PMS rate is 6.5 × 10^-5 ^per SNP base per meiosis, while the mutation rate has been estimated around 3 × 10^-10 ^per base per cell division [39,40]. Therefore, PMS may be a significant determinant of allelic inheritance and allele frequencies at the population level. Finally, our approach for measuring PMS can be extended to other environmental conditions and to strains with genetic perturbations for the genome-wide study of meiotic recombination and mismatch repair.

## Materials and methods

### Strains, media and cell dissection

The heterozygous diploid parental strain (*MATa/MATα ho/ho::hisG +/lys2 +/lys5 gal2/gal2*) was the result of mating strains S96, which is isogenic to S288c [19], and YJM789 [18]. Sporulation of the parental strain was induced by transferring an overnight YEPD culture to SPS liquid medium [41], letting it reach an OD_600 _of 1.4 and then transferring it to double the volume of 1% potassium acetate. All cultures were grown by vigorous shaking at 30°C. After 2 days of incubation in potassium acetate, tetrads were dissected in YEPD plates and the resulting spores were allowed to germinate at 30°C. Spores were constantly monitored to spot the first mitotic cell division. At the two-cell stage, the mother or daughter cell was further separated under the dissection microscope. Finally, all cells were grown to colonies at 30°C for 2 days [29,42-44].

### DNA extraction and hybridization

Each of the eight mother and daughter cells from four tetrads were independently cultured overnight at 30°C in 100 ml of YEPD liquid medium. The four tetrads selected here had not been previously genotyped in [16]. DNA was extracted from each culture using a QIAGEN (Hilden, Germany) Genomic Tip following the manufacturer's protocol. DNA (10 μg) was fragmented using DNase I and 3' biotin-labeled using an Affymetrix (Santa Clara, California, USA) kit. Hybridization was finally performed as described previously [16]. The Affymetrix tilling microarray used is a custom microarray that interrogates the genomic sequences of both strains, S288c and YJM789, at 4-bp resolution [16].

### Genotyping and recombination event annotation

In [16,25], a semi-supervised genotyping approach was advantageous due to the large number of segregant arrays (*n *> 200). Here, the total number of PMS arrays was smaller (*n *= 32), so we chose to assign genotypes in a supervised fashion, using a likelihood ratio based on the multivariate log-intensity distributions learned from the arrays in [16,25]. To ensure compatibility, we first used VSN [45] to normalize probe intensities from the PMS arrays to a reference distribution computed from the arrays in [16,25].

Probe sets identified as likely to produce excess genotyping error in [16,25], and consequently omitted there, were dropped for the PMS arrays as well. Individual genotype calls for the PMS arrays were further filtered as in [16,25]: log-intensity vectors that were either too close to the decision boundary (log-likelihood ratio scores below 36.7 in absolute value) or were outliers relative to their assigned genotype distribution (Mahalanobis distance to the appropriate genotype centroid greater than 5) were not assigned a genotype. For further details concerning the genotyping method and filtering, please see references [16,25].

After splitting the eight mother and daughter cells arising from a single meiosis into two pseudo-tetrads, recombination event annotation was done using a combination of automatic and manual annotation steps as previously described [16]. For computation, observed complex COs and NCOs with discontinuous gene conversion tracts were not distinguished from other COs or NCOs. Markers with discordant genotype calls for the mother and daughter cells arising from a single segregant were deemed to be PMS events.

### Calculating the probability of observing terminal and adjacent PMS events by chance

The likelihood that adjacent PMS markers occurred by chance depends in a complex way on the full configuration of recombination events. Using the observed configuration of recombination events containing three or more markers and at least one PMS event, we performed two simulations. First, this collection of events was observed to contain 32 PMS markers. To assess whether PMS markers' apparent preference for the ends of recombination events could be due to chance, we assigned 32 simulated PMS markers uniformly at random. (For COs, simulated markers were allowed to fall on either of the two involved strands.) We then computed the fraction of simulated recombination events with a terminal marker. In 1,000 simulations, this fraction was always far below the observed fraction (Figure S2 in Additional file 4).

Next, we performed a second simulation to assess the observed occurrence of multiple PMS markers at consecutive positions within a single recombination event. Given the results of the first simulation, we now hypothesized that a substantial fraction (76.9%) of PMS markers must be terminal, but that the remainder are uniformly distributed within the interiors of the intervals. In 1,000 repetitions of this second, non-uniform sampling scheme, we only once found two simulated recombination events with multiple, consecutive terminal markers; we never found three such events.

### Data deposition

The array data have been deposited in the ArrayExpress database (accession number E-TABM-1031).

## Abbreviations

Bp: base pair; CO: crossover; DSB: double-strand break; indel: insertion or deletion; MMR: mismatch repair; NCO: non-crossover; PMS: post-meiotic segregation; SNP: single nucleotide polymorphism.

## Authors' contributions

EM and LMS designed the study, EM performed the experiments, RB and EM analyzed the data, LMS and WH provided suggestions for data analysis, and all authors co-wrote the manuscript.

## Supplementary Material

Additional file 1 **Markers where post-meiotic segregation has been inferred**. The first_S288c and last_S288c columns correspond to the chromosomal coordinates of the marker using the S288c coordinate system. The rest of the headers give the spore number, the chromosome (chr), the S288c:YJM789 segregation ratio and the S288c and YJM789 genotype of the marker. Click here for file

Additional file 2 **Marker coordinates and genotype calls for all spores**. The first, last and chr columns are as described in the legend of Additional file 1. The type column contains S for SNPs, I for insertions, D for deletions, and M for probe sets that interrogated more than one polymorphism (that is, consecutive SNPs, or a SNP near an insertion or deletion). Apart from those columns there is one column per genotyped cell, the header of which is composed of "wt", the tetrad number (1, 5, 7, 8), the mother or daughter cell (1,2) and the spore (a, b, c, d), each separated by an underscore. A genotype call of 1 corresponds to S288c. Click here for file

Additional file 3 **Inferred CO and NCO interval locations**. The first_S288c and last_S288c columns correspond to the maximal method described in [16], in which the tract is defined by the two nearest unconverted markers. In the type column, × denotes a CO and C denotes an NCO as described in [16]. The pms column denotes whether a PMS event was observed in a given recombination interval. Other column headers give tetrad number and spore letter. Click here for file

Additional file 4 **Supplementary figures**. Figure S1: position of PMS markers within recombination tracts. Each row corresponds to a single recombination event and each vertical segment depicts a marker involved in such an event. Markers where PMS occurred are shown as larger red segments. Indel markers are shown as rectangles instead of segments. Vertical axis labels give tetrad, chromosome, and first base of the recombination event. For the event wt_7 chr06:74792, also depicted in Figure 2b, only one conversion tract is shown. Figure S2: PMS tends to occur at the ends of conversion tracts. The figure shows the degree to which there is overrepresentation of events with PMS markers exactly at one end or the other of the conversion tract. In 1,000 simulations, the fraction of recombination events with one or more terminal PMS markers was recorded (see Materials and methods). The histogram shows the distribution of these fractions; the blue vertical line shows the observed fraction of events with terminal PMS events. Figure S3: Events where more than one PMS marker was observed. Four recombination events had more than one marker where PMS occurred. Two of these events are depicted in Figure 2 in the main text and the other two are illustrated here: **(a) **a CO in chromosome II; and **(b) **a NCO in chromosome IX. As in Figure 2, red/blue vertical segments represent markers with the S288c/YJM789 genotype along the chromosomes of the two mother and daughter cells resulting from the first mitosis of each spore (A, B, C and D). Horizontal black lines indicate inferred NCOs, and the diagonal, inferred COs. Green vertical segments immediately on top of the coordinate axis denote markers where PMS occurred and orange segments denote markers with non-Mendelian segregation. Figure S4: SNP distribution along gene conversion tracts. For each recombination interval, markers were assigned to the fraction of the interval they spanned. For example, in a one-marker interval, the one and only marker was assigned to the full range from 0% to 100%; for a three marker interval, the first marker was assigned to 0% to 33%, the second to 33% to 66%, and the third to 66% to 100%, and so on. Non-SNP markers were ignored. The frequency with which any position corresponded to a particular SNP type was then computed over the full range of 0% to 100%. No SNP type appears to show positional bias. Click here for file
