# Supplementary figures and images for: Genome-wide survey of post-meiotic segregation during yeast recombination
Source: Genome Biol. 2011 Apr 11;12(4):R36. doi: 10.1186/gb-2011-12-4-r36 (PMC3218862; doi:10.1186/gb-2011-12-4-r36)

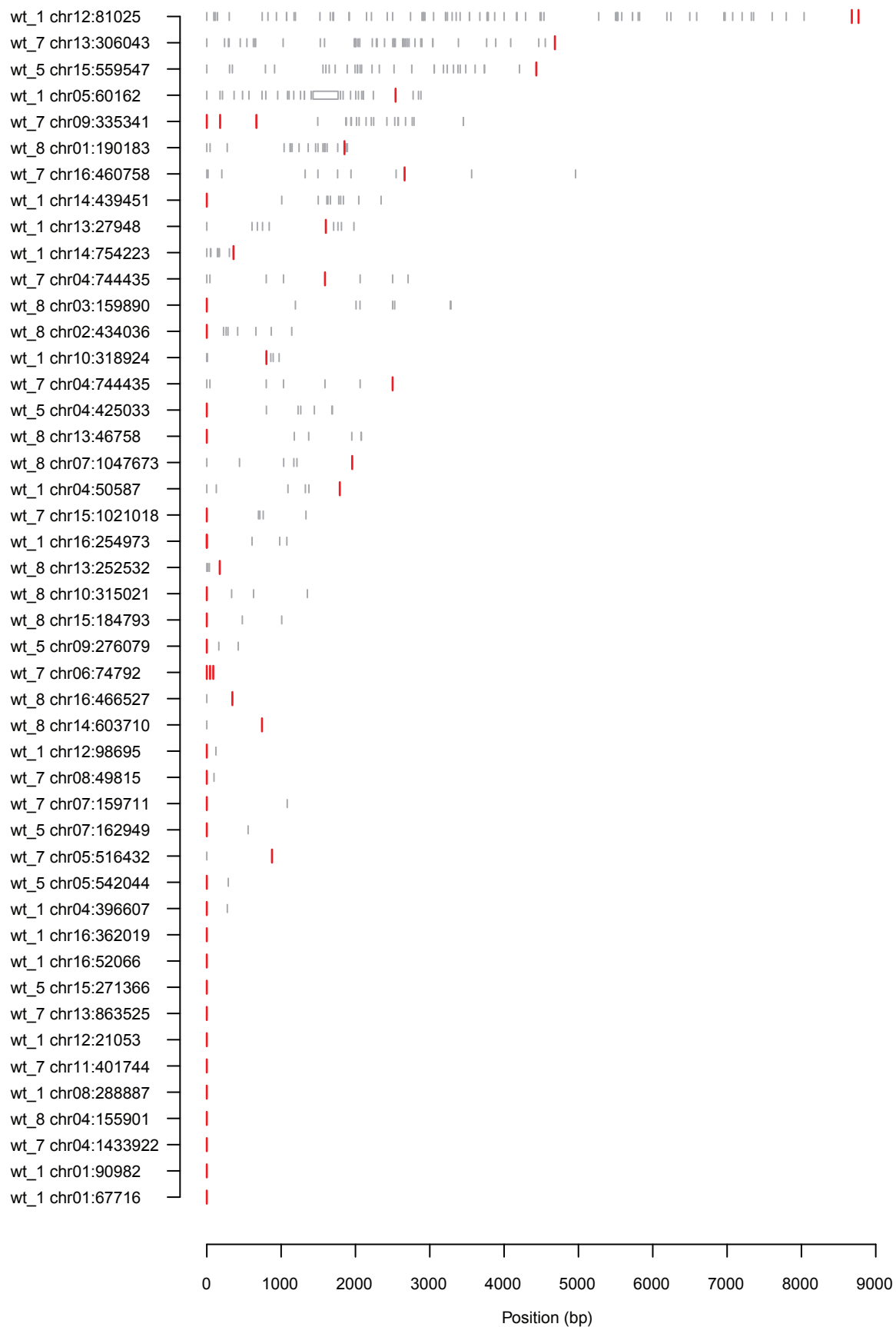

**Figure S1**

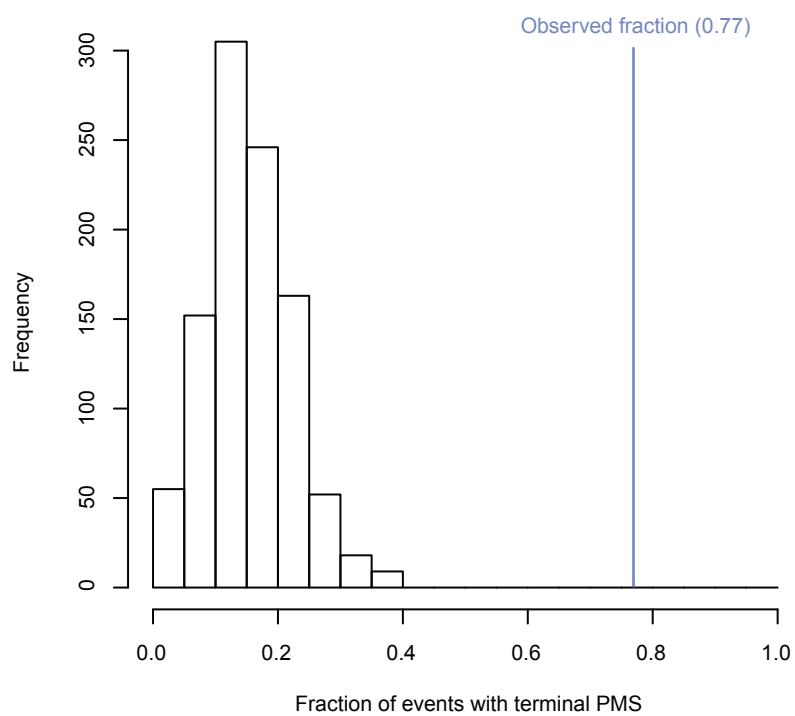

**Figure S2**

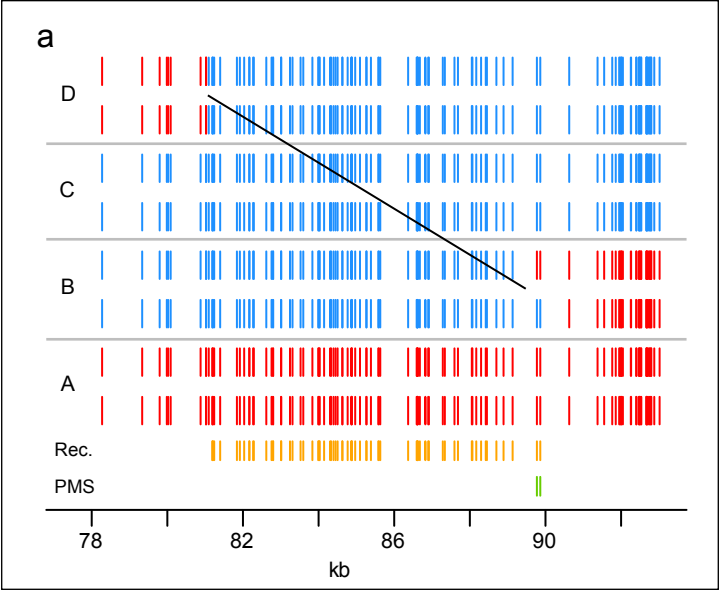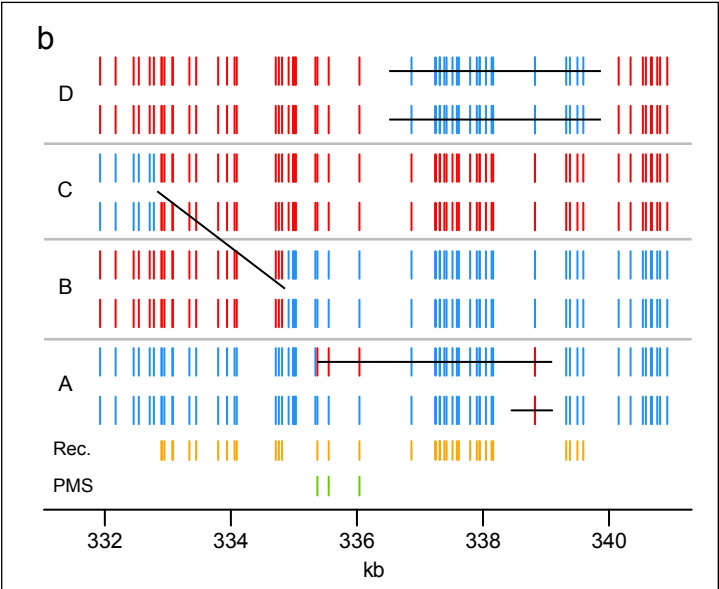

**Figure S3**

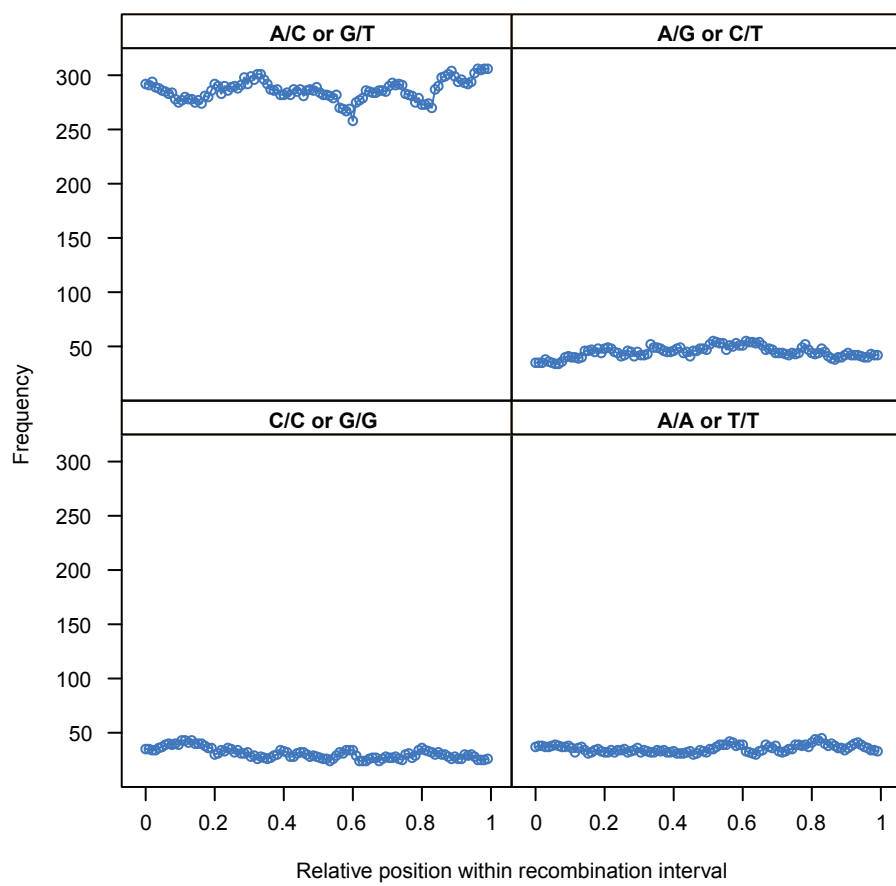

**Figure S4**

Supplement: Additional file 4 — Supplementary figures. Figure S1: position of PMS markers within recombination tracts. Each row corresponds to a single recombination event and each vertical segment depicts a marker involved in such an event. Markers where PMS occurred are shown as larger red segments. Indel markers are shown as rectangles instead of segments. Vertical axis labels give tetrad, chromosome, and first base of the recombination event. For the event wt_7 chr06:74792, also depicted in Figure 2b, only one conversion tract is shown. Figure S2: PMS tends to occur at the ends of conversion tracts. The figure shows the degree to which there is overrepresentation of events with PMS markers exactly at one end or the other of the conversion tract. In 1,000 simulations, the fraction of recombination events with one or more terminal PMS markers was recorded (see Materials and methods). The histogram shows the distribution of these fractions; the blue vertical line shows the observed fraction of events with terminal PMS events. Figure S3: Events where more than one PMS marker was observed. Four recombination events had more than one marker where PMS occurred. Two of these events are depicted in Figure 2 in the main text and the other two are illustrated here: (a) a CO in chromosome II; and (b) a NCO in chromosome IX. As in Figure 2, red/blue vertical segments represent markers with the S288c/YJM789 genotype along the chromosomes of the two mother and daughter cells resulting from the first mitosis of each spore (A, B, C and D). Horizontal black lines indicate inferred NCOs, and the diagonal, inferred COs. Green vertical segments immediately on top of the coordinate axis denote markers where PMS occurred and orange segments denote markers with non-Mendelian segregation. Figure S4: SNP distribution along gene conversion tracts. For each recombination interval, markers were assigned to the fraction of the interval they spanned. For example, in a one-marker interval, the one and only marker was assigne [file gb-2011-12-4-r36-S4.PDF]
